# Women's Choice of Positions during Labour: Return to the Past or a Modern Way to Give Birth? A Cohort Study in Italy

**DOI:** 10.1155/2014/638093

**Published:** 2014-05-15

**Authors:** Salvatore Gizzo, Stefania Di Gangi, Marco Noventa, Veronica Bacile, Alessandra Zambon, Giovanni Battista Nardelli

**Affiliations:** ^1^Department of Woman and Child Health, University of Padua, 3 Giustiniani Street, 35128 Padua, Italy; ^2^Dipartimento di Salute della Donna e del Bambino, U.O.C. di Ginecologia e Ostetricia, Via Giustiniani 3, 35128 Padova, Italy

## Abstract

*Background*. Childbirth medicalization has reduced the parturient's opportunity to labour and deliver in a spontaneous position, constricting her to assume the recumbent one. The aim of the study was to compare recumbent and alternative positions in terms of labour process, type of delivery, neonatal wellbeing, and intrapartum fetal head rotation. *Methods*. We conducted an observational cohort study on women at pregnancy term. Primiparous women with physiological pregnancies and single cephalic fetuses were eligible for the study. We considered data about maternal-general characteristics, labour process, type of delivery, and neonatal wellbeing at birth. Patients were divided into two groups: Group-A if they spent more than 50% of labour in a recumbent position and Group-B when in alternative ones. *Results*. 225 women were recruited (69 in Group-A and 156 in Group-B). We found significant differences between the groups in terms of labour length, Numeric Rating Scale score and analgesia request rate, type of delivery, need of episiotomy, and fetal occiput rotation. No differences were found in terms of neonatal outcomes. *Conclusion*. Alternative maternal positioning may positively influence labour process reducing maternal pain, operative vaginal delivery, caesarean section, and episiotomy rate. Women should be encouraged to move and deliver in the most comfortable position.

## 1. Introduction

For a long time, positions during labour could be freely changed or modified according to parturient desires.

Unfortunately, in developed countries the hospital admission of labouring women leads obstetrical practice to restrain spontaneous and instinctive attitude and to focus strictly on intrapartum fetal wellbeing and maternal comorbidities [1, 2].

This way, the parturient receives fewer opportunities to labour and deliver in a preferred position, assuming the recumbent one as standard because of its easier monitoring of fetal wellbeing, administration of intravenous therapy, loco-regional anaesthesia, and performance of medical procedures, perineal support, and birth assistance [2, 3].

The effects of different maternal positions during labour on maternal-fetal and neonatal outcomes are rarely in agreement and available evidences in this field are often controversial and fragmentary [1, 4, 5].

The vertical positions may benefit from “gravity effect” potentially able to reduce aortocaval compression, to make uterine contractions effective and to favour a better fetus alignment in the birth canal and to increase pelvic outlet diameters, reducing intrapartum maternal and neonatal complications [6–9].

Anyway counterparts evidences reported an increased haemorrhagic risk associated with upright positions [1, 10–12] due to more perineal damage than uterine atony (often requiring medical and surgical procedures and potentially impairing future pregnancy planning and chances) [13–15].

Certainly, recumbent position makes it easier to palpate the mother's abdomen in order to monitor contractions, to perform vaginal examinations and invasive manoeuvres, to check the fetal head position, and to assess the fetal heart rate.

Conversely, because of increased risk of maternal abdominal blood vessels compression, less effectiveness of uterine contractions, less perineal muscle relaxation, high rate of analgesia request, and long labour length, recumbent position seems associated with more operative deliveries, severe pain [1, 3, 16], abnormal fetal heart trace, and greater episiotomy rate [1, 10, 11, 17, 18].

Last but not least, since intrapartum complications are frequently reported when fetal occiput is in posterior position (OP), some authors investigated if maternal labouring position may have a role in facilitating spontaneous rotation to occiput anterior position (OA) without conclusive evidences [19–21].

The aim of our study was to compare patients spending in a recumbent position more than 50% of labour to those assuming a preferred alternative position (vertical position) in terms of intrapartum, maternal/fetal, and neonatal outcomes. The second aim of the study was to establish if differences exist among two groups in terms of fetal head rotation rate from OP to OA.

## 2. Methods

We conducted an observational cohort study on pregnant women admitted to the delivery room of University of Padua, Woman and Child Health Department, in the interval time between January 2013 and December 2013.

All the enrolled patients have been properly informed about the aim of the study and they consented to the usage of their data according to the Italian law (675/96).

We considered primiparous women with uncomplicated pregnancies and single fetuses in cephalic presentation before or at the onset of labour eligible for the study.

According to the defined criteria, labour onset was defined by regular uterine contractions and cervical dilatation of at least 2 cm; the second labour stage was defined when a full dilatation of the cervix is attained [22].

We considered inclusion criteria to be as follows: age more than 18 years, BMI between 18 and 30 Kg/m^2^, assessment of occiput fetal position at the labour onset and confirmed by ultrasound [23], and information about intrapartum analgesia administration. We excluded all cases of vaginal delivery in a previous cesarean section, cephalic fetal presentation after manual rotation of the fetus from OP to OA, labour induction, and augmentation.

General data of parturients were collected in an electronic service at recovery. Data about intrapartum care were collected through Friedman labour partogram and midwifery records. One of the authors, supported by the midwifery (V.B.), collected data daily and reported them in an excel database.

For all women, data about maternal general characteristics (age, BMI, and gestational age), labour process (length of first and second stages of labour, fetal occiput position at the labour onset and at birth, mean value of Numeric Rating Scale (NRS) score detected during labour and before analgesia administration when required, and analgesia request rate), mode of delivery (spontaneous, operative vaginal delivery or emergent caesarean section (CS), need of episiotomy, and rate of perineal tears in cases of vaginal deliveries), and neonatal wellbeing at birth (Apgar score at 5th, and fetal pH value at birth) were recorded.

Patients were included in Group-A when they spent more than 50% of their labour in recumbent position (supine or lateral) and in Group-B when they preferred an alternative position (upright, squatting, sitting on the ball, or “on all fours” position).

All eligible patients received exclusively a midwifery intrapartum care, except for urgent CS or operative vaginal deliveries cases. All eligible patients assumed a spontaneous position without any medical or midwifery prescription. Regarding the analgesia, all women received epidural analgesia, when required, without the use of opioid.

Maternal positions were considered as follows.

- Recumbent position: the pregnant is lying on her back, above the bed at an angle up to 45 degrees, or on her side preferring that one on which the fetal back and the occiput are located. A pillow between the legs (extended or flexed) was allowed.
- Upright position: the woman is in an upright position standing by herself or against to a support (bed, chair, or partner).
- Squatting position: the patient crouches during contraction and then recuperates during relaxation.
- Sitting position: the pregnant is sitting on a bed, on a chair, or on a ball.
- Position “on all fours”: the pregnant is kneeling and bent forward in order to support her weight with arms.

The primary endpoint of the study was to collect possible differences between two groups in terms of labour process, type of delivery, and neonatal wellbeing.

The secondary endpoint was to establish if differences exist among two groups (Group-A versus Group-B) in terms of fetal head rotation rate from OP to OA and into Group-B which is the best alternative position.

Statistical analysis was performed using SPSS (Chicago, IL) software for Windows version 18. We performed the Kolmogorov-Smirnov to test normality of distribution. Continuous data have been tested with the *t*-test, and categorical variables have been tested with the *χ* ^2^ test or Fisher's exact test when appropriate. Statistical significance was defined as *P* < 0.05.

## 3. Results

In the considered interval time, 225 women were eligible for the study. Among them, 69 patients joined Group-A and 156 joined Group-B. In detail, Group-B patients assumed the upright position in 46.1% of the cases, the sitting position in 21.1% of the cases, the “on all fours” position in 16.2% of the cases, and a balloon-squatting position in 16.6% of the cases.

Mean age was 31.13 ± 6.1 years (ranging 18–44 years), mean BMI was 23.59 ± 3.5 Kg/m^2^ (ranging 18–30 Kg/m^2^), and mean gestational age was 39.2 ± 1.2 weeks (ranging 37–41 weeks). Group-A and Group-B were homogenous for general maternal characteristics (Table 1).

**Figure 1:**
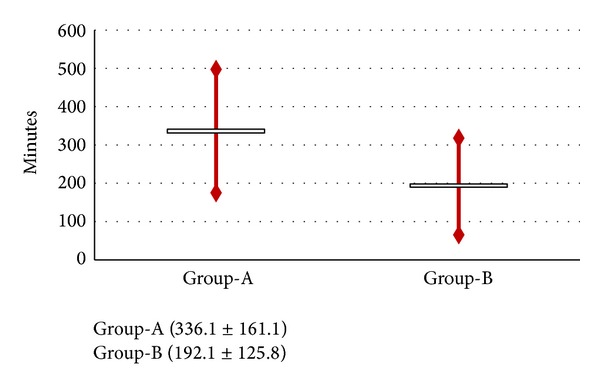
Comparison between the groups (Group-A versus Group-B) in terms of length of first stage of labour.

**Figure 2:**
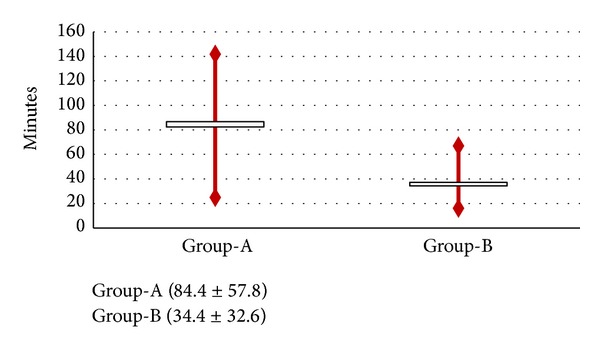
Comparison between the groups (Group-A versus Group-B) in terms of length of second stage of labour.

**Figure 3:**
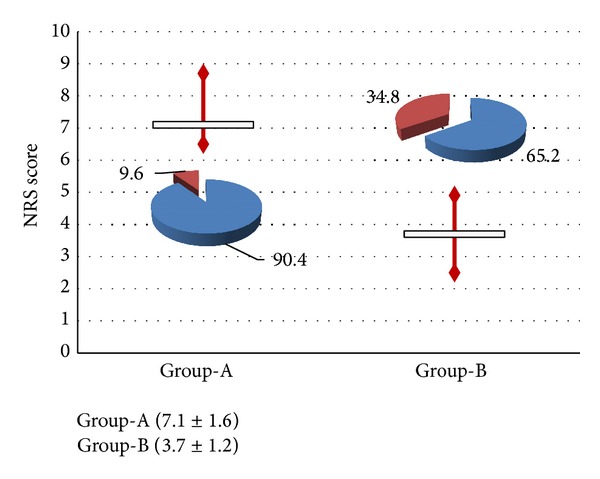
Comparison between the groups (Group-A versus Group-B) in terms of intrapartum NRS score and epidural analgesia request rate.

**Figure 4:**
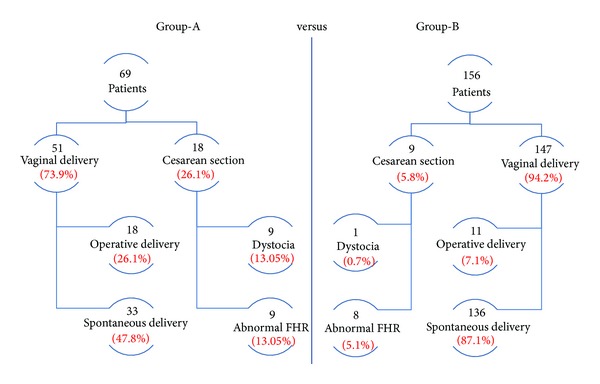
Flow diagram reporting type of delivery and indication of intrapartum caesarean section (comparison between the groups: Group-A versus Group-B).

Data about labour process, intrapartum pain and analgesia request, type of delivery, and neonatal outcome are detailed, reported in Table 1 and Figures 1–4.

Significant statistical differences were found in the length of both first and second labour stages (mean value of 336.1 ± 161.1 versus 192.1 ± 125.8; 84.4 ± 57.8 versus 34.4 ± 32.6 minutes, resp.; *P* < 0.001) (Figures 1-2; between two groups (Group-A versus Group-B)).

Similarly, significant differences in terms of pain level with a mean NRS score of 7.1 ± 1.6 versus 3.7 ± 1.2 were, respectively, detected (*P* < 0.001). The two groups significantly differed for the analgesia request rate, respectively, with 34.8% versus 9.6% rate (*P* < 0.0001) (Figure 3).

Regarding the mode of delivery, 47.8% of Group-A patients delivered by vaginal route, 26.1% required operative vaginal delivery, and 26.1% underwent CS.

Group-B patients delivered in 87.1% by vaginal route and required operative vaginal delivery in 7.1% and CS in 5.8% (*P* < 0.001) (Figure 4).

In Group-A, dystocia occurred in 13.05% of the cases and abnormal fetal heart rate in 13.05% of the cases while in Group-B this condition occurred, respectively, in 0.7% and 5.1% (*P* < 0.05) (Figure 4).

Episiotomy was performed in 100% of Group-A patients who delivered by vaginal route compared to the 32.7% of Group-B (*P* < 0.001), while 1st-2nd degree vaginal tears occurred, respectively, in 5.9% versus 49% of the cases (*P* < 0.001); no differences between two groups in terms of neonatal outcomes were reported.

Concerning the distribution of fetal occiput position at the labour onset, the OP rate resulted in being comparable in two groups with 40.6% in Group-A and 36.5% in Group-B (*P*: n.s.).

Considering OP cases (28 cases in Group-A and 57 cases in Group-B) a strong significant difference was found in terms of delivery outcome.

CS was necessary in 27 patients: 46.4% in Group-A compared to the 12.3% in Group-B (*P* < 0.0001).

Significant differences in terms of OP persistence at delivery were also found in those delivering vaginally: in Group-A patients, OP persisted till birth in 39.6% of the cases while in Group-B only in 28% of the cases (*P* < 0.001).

Considering only Group-B patients, no differences were found comparing alternative position for all the outcomes considered. Detailed data are summarized in Table 1 and Figures 1–4.

## 4. Discussion

A satisfying childbirth experience is influenced by women's self-control, labour pain perception, expectations, and health care support. The possibility to change the position in labour might positively influence childbirth experience and also the good course and outcome of labour [24].

Several advantages have been claimed for nonrecumbent labour, thanks to “gravity effect” on uterine perfusion, on contractions effectiveness, and on fetal alignment to the pelvic angles and diameters [18].

In the first stage of labour vertical positions seem associated with lower pain, reduced labour length, and perception of physiological event, resulting in an increased women's comfort and satisfaction after childbirth [25, 26].

These evidences have been confirmed in a recent meta-analysis revealing that vertical positions are also associated with a lower analgesia request and necessity of interventions [4].

However, all the existing studies did not provide a definitive message and were postponed to further investigation to define the real role of position in the labour process [1, 4, 5].

Although some authors reported no effect of maternal position on labour length [18, 27, 28] a significant reduction in length of both first and second labour stages was found in our patients assuming alternative positions and confirming a possible favouring effect of gravity in effective uterine contractions and fetal alignment to the birth canal. Episiotomy, operative vaginal delivery, and severe vaginal tears rate confirmed in our series of cases previous evidences regarding the positive effect of alternative position [9, 29–32].

This finding can be related to a better and gradual maternal perineum compliance to the fetal head descent, reducing anatomical and functional perineal damage and consequent dyssynergia.

Vertical positions are burned to more difficult medical management when peculiar conditions (amniotomy, oxytocin induction, fetal monitoring, and uterine contraction tracings) and interventions (epidural analgesia) are required and spontaneous movements and position change are not feasible [25].

Nevertheless, several studies reported that when a spontaneous analgesic and comfortable position is allowed, labouring women may benefit from a shorter labour length, avoidance of augmentation, and lower pain, reaching childbirth with strong motivation [7, 8, 16, 33]. In agreement with previous studies, a significantly lower analgesia rate was recorded when a vertical position was assumed, compared to the recumbent one (probably due to lower perineum reflex muscle contraction of upright position).

If on one hand intrapartum epidural analgesia is considered a safe and effective procedure [34, 35], on the other hand it represents an adjunctive cause of maternal hypomobilization during labour and an indirect risk cofactor for fetal malposition persistence [19].

Many authors postulated that intrapartum persistent OP position (general prevalence ranging from 2% to 13%) represents a risk factor for poor maternal and neonatal outcomes [19, 23, 36].

When a persistent OP occurs, different adverse obstetrical events are reported such as prolonged first and second labour stages, increased epidural analgesia request, higher risk of postpartum haemorrhage, increased CS, operative vaginal deliveries, and 3rd- and 4th-degree perineal tears rate [19, 23, 36].

Frequently these intrapartum conditions resulted in low Apgar score, neonatal trauma, acidemic cord blood gas concentrations, admission to neonatal intensive care unit, and newborn encephalopathy [19, 23, 36].

Different obstetrical manoeuvres have been proposed to facilitate the fetal head rotation from OP to OA (oxytocin augmentation and manual rotation), but none resulted in being more effective than maternal vertical position during labour [19, 20].

In a cohort of 225 parturients we found that vertical positions appeared helpful in foetal head rotation during labour, reducing the rate of operative vaginal deliveries and CS. Unfortunately, our sample did not allow us to discriminate which vertical position has to be preferred. Stremler et al. suggested that hands-and-knees positioning was more effective than others in reducing persistent back pain and favouring rotation of OP to OA but their study sample did not reach statistical power [37].

The upright position takes advantage of the gravity, increased size of the pelvic diameter, thanks to the nutation movement and to the coccyx retropulsion, the decline of the extreme cephalic, less painful and more effective contractions, pain relief for reduced pressure on the sacrum, increased confidence in the second labour stage, and lower perineum stretch. The sitting position takes advantage on gravity, on use of lumbar massage, and on an increased pelvic diameter with better fetal alignment to the pelvis, but it may increase the pressure on the sacrum with a major risk of perineal trauma. The “on all fours” position reduces the effect of gravity, the peak and duration of the contractions, and the pain due to a lower fetal pressure on the pelvis; it allows practicing the lumbar massage and favors the fetal internal rotation. This is the most recommended position to correct and prevent fetal malposition, to reduce cervical edema and the sacral pressure of the presenting part, and to increase the pelvic anteroposterior diameter in the expulsive phase. The squatting position allows using gravity, increasing the pelvic diameters and the counternutation for the fetal head descent, and strengthening the feeling of thrust and relaxation of perineal muscles [25, 38].

Our encouraging results about vertical positions need to be further confirmed by large cohort studies and do not solve the existing debate.

Unfortunately, nowadays the “gravity effect” able to increase the maternal perineum compliance to fetal progression should be considered a theoretical assumption. Considering available literature and in absence of clear and strong evidences, it seems reasonable not to impose on women a labouring posture different from the spontaneous one [21].

Although Golara et al. [39] suggested that the maternal immobilization during labour may increase the incidence of dystocia, when strict monitoring of fetal wellbeing [40] and high risk pregnancy medicalization [41–44] or intensive intrapartum care are necessary, the use of alternative positions should be carefully evaluated.

Our data on a series of uncomplicated pregnancies allows us to suggest that, in absence of prelabour or intralabour complications, the alternative vertical positions may positively influence labour process reducing maternal pain and operative vaginal delivery, CS, and episiotomy rate.

Our study is actually the first one assessing the role of maternal labouring vertical position in occiput rotation from OP to OA demonstrating its real benefit on labour process and delivery.

Although further studies in this field are mandatory and most theoretical speculations need to be clarified, in absence of prepartum/intrapartum maternal-fetal complications, all women should be encouraged to move and to deliver in the most comfortable position, preferring a vertical position when OP is diagnosed.

## Figures and Tables

**Table 1:** Comparison between the groups (Group-A versus Group-B) in terms of maternal, labour, and neonatal characteristics (*Kolmogorov-Smirnov tests showed a normal distribution of continuous variables*).

Maternal, labour, and neonatal characteristics			
Variables	Groups (number)	Mean (±standard deviation)	*P* value
Maternal age (years)	Group-A (69)	31.83 (5.55)	n.s.
	Group-B (156)	30.83 (6.36)	
	Total (225)	31.13 (6.13)	

Gestational age at birth (weeks)	Group-A (69)	39.70 (1.40)	n.s.
	Group-B (156)	39.02 (1.01)	
	Total (225)	39.23 (1.18)	

BMI	Group-A (69)	24.26 (3.53)	n.s.
	Group-B (156)	23.29 (3.57)	
	Total (225)	23.59 (3.58)	

Intralabour pain (Numeric Rating Scale score)	Group-A (69)	7.1 (1.6)	*P* < 0.001
	Group-B (156)	3.7 (1.2)	
	Total (225)	4.72 (2.1)	

First stage labour length (minutes)	Group-A (69)	336.1 (161.1)	*P* < 0.001
	Group-B (156)	192.1 (125.8)	
	Total (225)	230.2 (149.9)	

Second stage labour length (minutes)	Group-A (69)	84.4 (57.8)	*P* < 0.001
	Group-B (156)	34.4 (32.6)	
	Total (225)	47.1 (45.9)	

Variables	Groups (number)	Number (percentage)	*P* value

Analgesia request	Group-A (69)	24 (34.8)	*P* < 0.0001
	Group-B (156)	15 (9.6%)	
	Total (225)	39 (17.3)	

Occiput posterior position at labour onset	Group-A (69)	28 (40.6)	n.s.
	Group-B (156)	57 (36.5)	
	Total (225)	85 (37.8)	

Persistent occiput posterior position at delivery (except cesarean sections)	Group-A (51)	11/28 (39.6)	*P* < 0.001
	Group-B (147)	16/57 (28)	
	Total (198)	27/85 (31.7)	

Apgar 5th minute <7	Group-A (69)	0 (0)	n.s.
	Group-B (156)	0 (0)	
	Total (225)	0 (0)	

pH < 7.2 at birth	Group-A (69)	9 (13)	n.s.
	Group-B (156)	30 (19.2)	
	Total (225)	39 (17.3)	
